# The Role of Oxidation Pattern and Water Content in the Spatial Arrangement and Dynamics of Oxidized Graphene-Based Aqueous Dispersions

**DOI:** 10.3390/ijms232113459

**Published:** 2022-11-03

**Authors:** Anastassia Rissanou, Ioannis Karnis, Fanourios Krasanakis, Kiriaki Chrissopoulou, Konstantinos Karatasos

**Affiliations:** 1 Department of Chemical Engineering, University of Thessaloniki, P.O. Box 420, 54124 Thessaloniki, Macedonia, Greece; 2 Department of Mathematics and Applied Mathematics, University of Crete, GR-71409, 70013 Heraklion, Crete, Greece; 3 Institute of Electronic Structure and Laser, Foundation for Research and Technology-Hellas, 70013 Heraklion, Crete, Greece; 4 Department of Chemistry, University of Crete, 70013 Heraklion, Crete, Greece

**Keywords:** graphene oxide, aqueous dispersion, clustering, counterion dynamics, simulations

## Abstract

In this work, we employ fully atomistic molecular dynamics simulations to elucidate the effects of the oxidation pattern and of the water content on the organization of graphene sheets in aqueous dispersions and on the dynamic properties of the different moieties at neutral pH conditions. Analysis of the results reveals the role of the oxidation motif (peripherally or fully oxidized flakes) in the tendency of the flakes to self-assemble and in the control of key structural characteristics, such as the interlayer distance between the sheets and the average size and the distribution of the formed aggregates. In certain cases, the results are compared to a pertinent experimental system, validating further the relevant computational models. Examination of the diffusional motion of the oxidized flakes shows that different degrees of spatial restriction are imposed upon the decrease in the water content and elucidates the conditions under which a motional arrest of the flakes takes place. At constant water content, the structural differences between the formed aggregates appear to additionally impart distinct diffusional characteristics of a water molecule. A detailed examination of the counterion dynamics describes their interaction with the oxidized flakes and their dependence on the water content and on the oxidation pattern, offering new insight into the expected electrical properties of the dispersions. The detailed information provided by this work will be particularly useful in applications such as molecular sieving, nanofiltration, and in cases where conductive membranes based on oxidized forms of graphene are used.

## 1. Introduction

Oxidized carbon-based materials are known to form macroscopically stable dispersions in aqueous media, even without the presence of dispersion agents [1,2]. The characteristics of such dispersions are known to decisively affect the physical properties of these mixtures [3,4,5,6,7]. The degree of hydration and the nature of the interactions among the components play a crucial role in the relative arrangement of the solute molecules and thus in the final structure formed in the dispersion [8,9]. Electrostatic [1], as well as specific, interactions (such as hydrogen bonding [10,11,12,13]) between the solute and the water molecules, in conjunction with the degree of hydrophilicity/hydrophobicity [13,14,15] of the oxidized carbon components or the presence of functional groups [15,16,17], essentially drive the self-assembly process of the carbon-based molecules. In the case of graphene oxide (GO) aqueous systems, the overall degree of oxidation [12,13], the presence or absence of specific oxidized groups (e.g., hydroxyl, epoxy, carboxyl) [18,19,20], the oxidation pattern (i.e., the spatial distribution of these groups) [12,13,21,22], and the charging state of the flakes [1,3,13] are directly associated with the self-organization process [23] and with the morphological features (average size, aggregation number, stacking behavior) of the formed aggregates [7,24,25].

The formation of spatially isolated or interconnected GO aggregates may significantly affect the properties of these systems [25,26], while the details of the inter- and intra-cluster arrangement (i.e., the average separation between the GO sheets belonging in the same or in different aggregates [27,28,29]), as well as the diffusional dynamics of entrapped water or ionic moieties within their interior [28,29,30,31], essentially determine the suitability of these systems as components in several industrial (e.g., in selective gas separation [32,33,34], desalination [16,35,36,37]), environmental (e.g., nanofiltration [38,39,40], water purification [17,39,41,42]), and biomedical (e.g., drug and gene delivery [43], toxicity assessment [44]) processes. Controlling these features by obtaining detailed information at the microscopic (or even better at the atomic) level is therefore essential for a rational design of these materials.

To this end, atomistic molecular simulations have emerged as a unique tool that offers high spatial and temporal resolution for the study of structural and dynamic properties of such systems [20,29,35,45,46,47,48,49,50,51]. The detailed information obtained by computational methods combined, where possible, with appropriate experimental techniques can lead to a better understanding of the mechanisms which operate at the microscopic level and which are responsible for the physicochemical behavior of the systems under investigation [38,48,49,52,53,54,55].

In this work, we employ atomistically detailed molecular dynamics (MD) simulations to study the effects of the different water content and of two distinct oxidation patterns of the GO flakes at neutral pH conditions on their spatial organization and on the characteristic transport properties of the dispersion constituents. We study the associative behavior of the flakes, the structural characteristics of the formed clusters, and the dynamics of the different components of the dispersions in a way not yet discussed in the literature. In addition, our analysis offers new insight into the role of the counterions in the self-assembly process of the flakes, thus providing new routes toward the control of key physical properties of these materials. Furthermore, the results of the simulations for the most commonly met oxidation pattern studied in this work are validated by comparison with experimental findings from a sample specifically designed to match the structural features of the simulated models.

## 2. Results

### 2.1. Experimental Results

The X-ray photoelectron spectroscopy (XPS) measurements performed showed that the atomic ratio of oxygen in the examined sample was approximately 17.5% with respect to the carbon, rendering a carbon-to-oxygen ratio of 4.8/1, very close to the 4.94/1 ratio considered for the GO-based flakes that were investigated computationally (see Table 1).

From the titration experiments (see Figure S1) it is clear that the sample shows a multi-step ionic behavior with three distinct pKα’s. The onset of a buffering can be observed at pH values 6.2–6.4 which can be attributed to the protonation of the carboxyl groups. The presence of another smaller step is observed at higher pH which can be attributed to the protonation of the hydroxyl groups. The pKα in this case is close to 9.7; however, this value cannot be accurately defined, due to the very low concentrations of the specific protonated groups. These results are in good agreement with what is known for GO in the literature, where multiple pKαs are reported at pH~9.8 attributed to the protonation of the hydroxyl groups, at pH~6.7 attributed to the protonation of the carboxyl groups, and lastly, at lower pH~3.6 which is due to the protonation of the carboxyl groups having adjacent hydroxyl groups [56]. Usually, the dominant step is the one attributed to the protonation of the carboxyl groups at pH~6.7.

**Figure 1:**
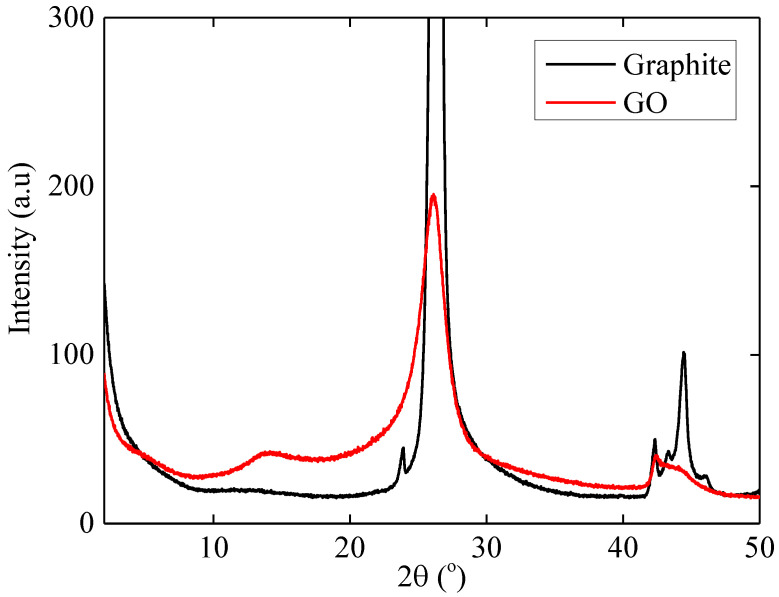
X-ray diffractogram of the synthesized graphene oxide sample. The measurement of graphite is included as well for comparison purposes.

Figure 1 shows the X-ray diffractogram of the graphene oxide that was synthesized utilizing the proper amount of oxidant for obtaining a degree of oxidation comparable to the simulated system together with the measurement of the pristine graphite.

The pristine graphite shows a main diffraction peak at 2*θ* = 26.5° which, according to Bragg’s law, corresponds to an interlayer distance of d~3.3 Å between the adjacent graphite sheets. As far as the graphene oxide is concerned, the partial oxidation of its sheets is evident by the appearance of a small peak at a lower angle, which indicates the insertion and bonding of oxygen species on the graphite sheets; however, the main diffraction peak of the material is still observed at the same angle with the one of graphite indicating the presence of unoxidized sheets. For the GO that has been oxidized utilizing 0.5 g of KMnO_4_, 2*θ* = 14.2 ± 0.6° corresponding to an interlayer distance d = 6.2 ± 0.3 Å. When a larger amount of oxidant is used for the synthesis of GO, lower diffraction angles and thus larger interlayer distances are attained at low water contents, accompanied by the disappearance of the graphite peak [57]. Further than the determination of the interlayer distance between the GO sheets, one can also calculate the average number of sheets that constitute each GO cluster. This can be calculated based on the Scherrer equation τ=Kλβcosθ, where *τ* is the crystallite size, *K* is a shape factor that equals 0.9, *λ* is the wavelength of the radiation, and *β* is the full width at half maximum of the peak. According to the Scherrer equation, the graphite clusters consist of ~70 layers, whereas a cluster of the partially oxidized GO studied here consists of approximately four sheets.

**Figure 2:**
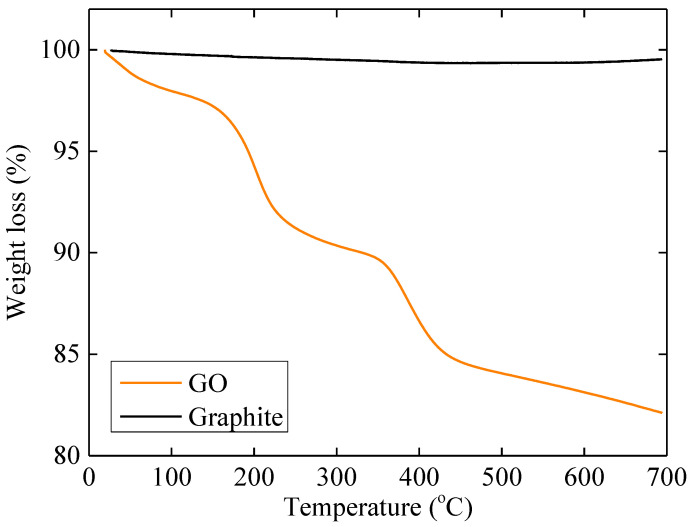
TGA measurements for the graphene oxide sample (GO) and pristine graphite.

Figure 2 shows the thermogravimetric (TGA) measurement of the synthesized GO. The initial drop observed at temperatures lower than 100° is attributed to the removal of water molecules that reside within the GO galleries (here, it is estimated to be close to 2% wt%). Between the temperatures 150 and 220 °C, the loss of mass is due to the decomposition of hydroxyl groups. In the temperature range between 350 and 450 °C, the mass loss is due to the removal of the most stable oxygen groups such as carboxyls and epoxides. Finally, above 450 °C, the small decrease in the mass of the sample observed can be attributed to the pyrolysis of the carbon skeleton of the flakes. At the same temperature range, no weight loss is observed for graphite, as anticipated by the absence of any functional group and its hydrophobicity that precludes adsorption of water.

Experimental measurements performed in the same sample after a period of more than 6 months reproduced the results with good accuracy. Therefore, no significant degradation of the GO sample was recorded within this period under the examined conditions and at the estimated water level [58].

### 2.2. Simulation Results

#### 2.2.1. Effects of the Oxidation Pattern on the Formed Morphology

Previous experimental and computational studies [23,26,59] have demonstrated that driven by the hydrophobic nature of the carbon atoms, graphene-based flakes tend to self-assemble forming aggregates, the morphology of which depends on parameters such as the degree of oxidation, the spatial distribution, and the nature of the oxidized groups and their charging state [13]. These parameters enter into the energetic interplay among the sheets, affecting hydrogen bond formation, the electrostatic, and the π-π interactions. As a result of these energetic factors but possibly due also to kinetic reasons [60], the graphene-based flakes may organize in clusters (i.e., aggregates) with characteristic size, aggregation number, and separation between the flakes. To study such morphological features and to elaborate more on the nanometer-scale structural characteristics of the dispersions, we employed appropriate analysis tools, examining measures such as the radial distribution function, rdf, *g*(*r*) [61] arising from the geometric centers of the flakes, as well as a pertinent clustering-detection algorithm.

**Figure 3:**
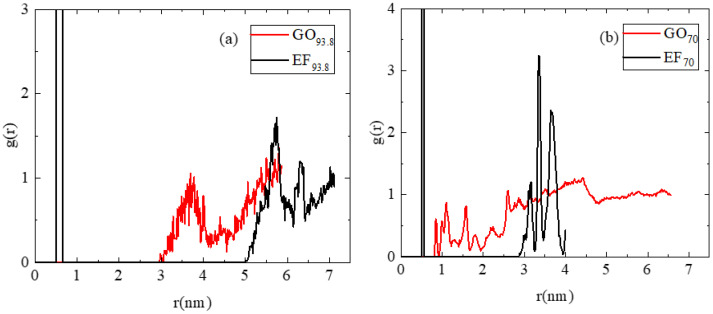
Radial distribution functions g(r) arising from the geometric centers of the graphene sheets for two GO and two EF-based systems at (**a**) 93.8 wt% and (**b**) 70 wt% concentration in water.

To determine the differences in the morphological characteristics of the graphene-based particles in the dispersions originating from the structural variations, we compared two pairs of systems based on the edge-functionalized (EF) and the graphene oxide (GO), with the same number of flakes and the same water content. This comparison highlights the effect of the different oxidation patterns. Results of the rdf analysis for two different water concentrations, i.e., 93.8 and 70 wt% are presented in Figure 3.

Focusing on systems EF_93.8_ and GO_93.8_ which bear the higher water content (Figure 3a), we observe distinct differences concerning the location, number, and width of the peaks. Since the radial distribution function is proportional to the probability of finding two particles (here the geometric centers of the flakes) at a separation *r*, these features provide information regarding the relative arrangement of the flakes. The rdf of the EF_93.8_ system is characterized by a sharp peak at a separation somewhat shorter than 1nm and several broader peaks at separations larger than 5 nm. The intense peak at a short separation indicates the presence of closely packed flakes. The wider and lower-amplitude peaks at separations longer than 5 nm indicate the presence either of individual flakes or groups of them, separated by larger distances. On the other hand, no close-packing arrangements are observed in the rdf spectra of the GO_93.8_ system. The curves are characterized by rather broad peaks of low intensity but at separations shorter compared to the low-intensity peaks observed in the EF_93.8_ system. The low intensity of the peak at ~2nm separation shows that the presence of flakes at very close proximity to other flakes is rare. These features are consistent with the presence of non-associated flakes or the formation of assemblies with a rather loose structure.

The decrease in the water content and increase in the number of flakes (Figure 3b) do not affect the main characteristics of the short-separation intense peak in the system EF_70_ which is found at 0.5 nm (i.e., indicating a persistent close-packed configuration), but it does result in a shift in the rest of the peaks at shorter distances and in an increase in their intensity and sharpness. The latter is consistent with a higher degree of localization of the groups formed by the flakes (since their relative separations become more “discretized”). Analogous changes in the spectral features of the GO_70_ system with respect to those of GO_93.8_ are also observed upon the decrease in the water content and the increase in the number of flakes per unit volume. At even lower water content (i.e., in systems GO_50_, GO_30_, GO_10_), the changes in the number, sharpness, and intensity of the peaks become even more pronounced (see Figure S2 in the supporting information). It is notable that at low water percentage (i.e., in the GO_30_ and the GO_10_ models), the peaks related to the first neighbors appear at a separation of 6.5 Å (practically independent from the water content), in good agreement with the experimentally determined interlayer distance of 6.2 ± 0.3 Å, as was described in Section 2.1.

The shift of the spectra to shorter distances and the increased sharpness of the peaks in both the EF and the GO-based systems imply that the presence of a larger number of flakes per unit volume and the higher viscosity due to the lower water percentage, promote the formation of more densely packed structures. However, qualitative and quantitative features of the flakes’ arrangement (number, location, intensity of the peaks) remain distinctly different between the EF and the GO-based systems. Calculation of the distribution of the inter-flake distances (see Figure S3) shows that for the EF flakes, they are centered at longer separations compared to those in the GO-based models, in line with the picture described from the rdfs; however, at lower water content, the width of the distribution for both the EF and the GO-based systems becomes broader.

**Figure 4:**
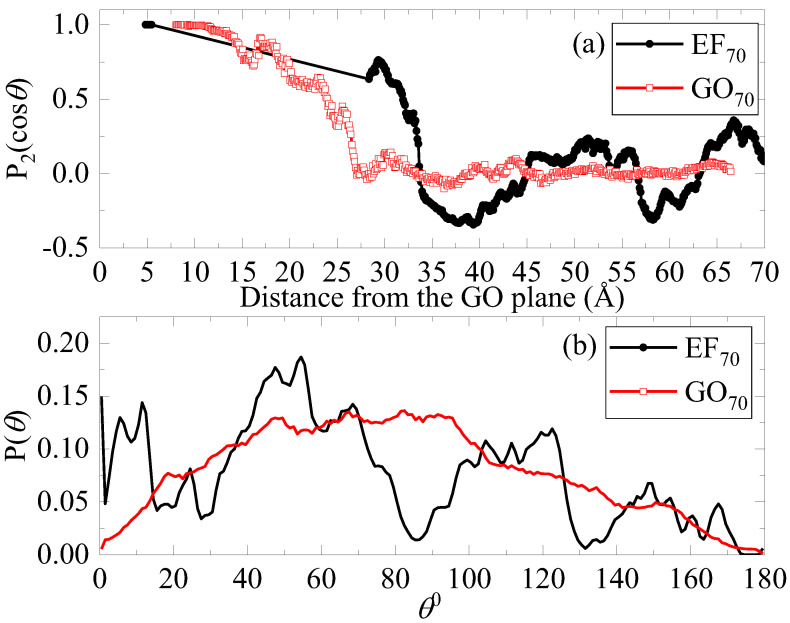
(**a**) The orientational order parameter of the flakes as a function of their separation, for systems comprising 56 flakes and at 70% wt% water content; (**b**) the corresponding angle distributions for the examined GO and EF-based systems.

To examine whether the flakes of the closely packed arrangement in the EF-based systems actually correspond to stacked configurations and to acquire more detailed information regarding the relative orientation of neighboring flakes at larger distances as well, we calculated the orientational order parameter quantified through the second Legendre polynomial, $P_{2}\left(\cos\theta \right)=\frac{3}{2}\langle \cos^{2}\theta \rangle -\frac{1}{2}$, where *θ* is the angle formed by the two vectors which are normal to the examined sheets (one sheet is taken each time as reference) [17]. This parameter assumes a value of 1 if a perfect alignment of the examined vector is detected with respect to the reference direction, a value of −0.5 when a perpendicular arrangement is detected between the examined vectors, and a value of 0 for a random orientation. We evaluated the orientational order parameter as a function of the separation between the flakes. Comparison between the systems with a different oxidation pattern and at the same concentration (i.e., 70 wt% in water) (for better statistical accuracy) is provided in Figure 4a. At very close distances, there are EF flakes that acquire a parallel orientation, i.e., forming stacked groups. Values of *P*_2_ above 0.5 at separations between 27 and 32 Å indicate a tendency for parallel orientation not associated with stacked configurations but either between well-separated flakes or between neighboring stacks. Beyond separations of 35 Å, the orientational parameter fluctuates around 0 but with rather large fluctuations. This behavior prompts a random orientation associated with groups of flakes (either stacked or not). For the GO flakes, the tendency for parallel orientation persists for moderately larger separations (i.e., up to approximately 15 Å), but that tendency is gradually lost, leading to randomly oriented flakes at longer distances. The different origin of the orientational behavior between the EF and the GO-based flakes is clarified from the behavior of the distribution of the orientational angle *θ*, as shown in Figure 4b. For EF sheets, *θ* attains almost “quantized” values, which prompts the presence of “interlocked” orientations. These may arise from individual or grouped (i.e., clustered) flakes that become spatially confined. For the GO flakes, the corresponding angle distribution is a continuous curve over the entire angle range, indicating that the GO sheets may assume any possible orientation.

**Figure 5:**
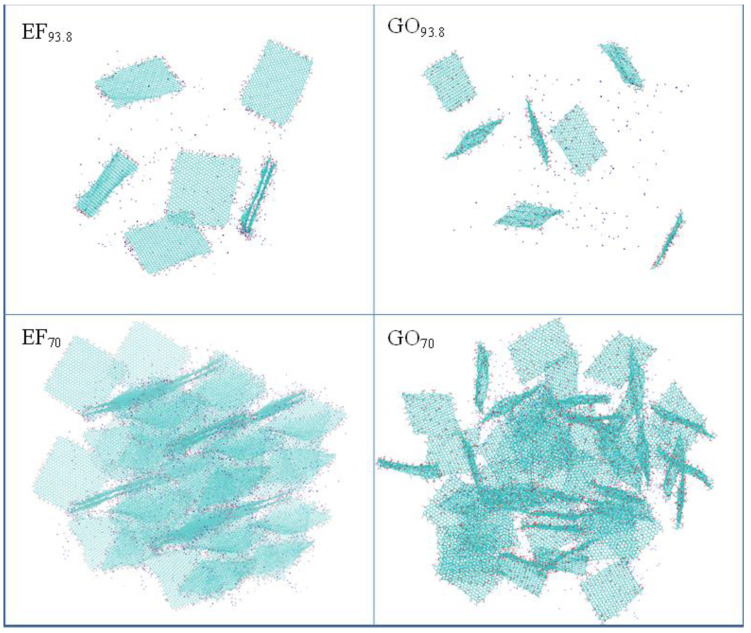
Snapshots of systems EF_93.8_ and GO_93.8_ (7 flakes at ~94 wt% in water) and EF_70_ and GO_70_ (56 flakes at ~70 wt% of water content). The graphene flakes are shown in dark cyan. Red dots represent oxygen atoms and blue dots the sodium counterions. Water molecules are omitted for clarity.

Figure 5 illustrates the two groups of EF and GO-based systems (i.e., GO_93.8_ and EF_93.8_ and GO_70_ and EF_70_). Although the pictures refer only to snapshots of the produced trajectories, one can already distinguish several of the features described by the statistical measures presented so far. For instance, the presence of stacked configurations is discernible in both the EF_93.8_ and the EF_70_ systems. Such closely packed configurations are prohibited in the GO_93.8_ and GO_70_ systems, due to the presence of the in-plane oxidized groups. In the latter, at the lower water content (GO_70_), the presence of almost parallel flakes can also be discerned but with a larger separation between them when compared to that observed in the EF-based systems. The associative characteristics of the flakes in the systems based on the EF or the GO sheets are quite different as shown in the snapshots and as implied by the analysis presented so far. To quantify the differences in their associative behavior, we examined the clustering characteristics of the two kinds of flakes using a previously described algorithm [62]. This algorithm estimates the number of sheets (N_s_) included in the formed clusters, based on a search radius around each sheet and the inclusion or exception of each neighbor from the cluster, depending on whether it has been previously included in a cluster or not. The search radius was extended up to the distance at which all the peaks appearing in the respective rdfs of the geometrical centers of mass are included (see Figure 3 and Figure S2), i.e., 6.5 nm for the EF and 5.5 nm for GO sheets. The estimation of the clusters’ occupancy was based on these criteria, irrespective of the relative orientation of the flakes.

**Figure 6:**
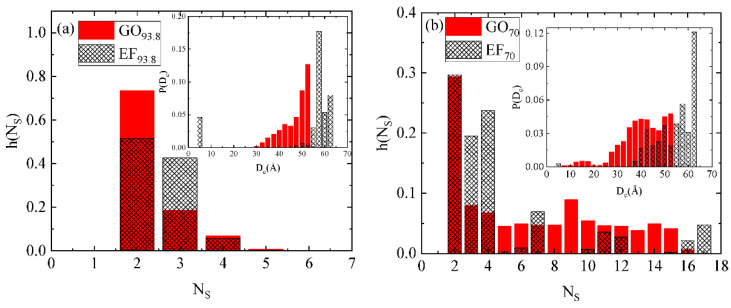
The probability distribution of the number of oxidized graphene flakes in a cluster for GO and EF systems of the same concentration (**a**) 93.8 wt% and (**b**) 70 wt% in water. (Insets): The corresponding probability distributions for the distances between pairs of flakes in a cluster.

The probability distribution of the number of graphene flakes in a cluster, h(s), is presented in Figure 6a,b, for the systems EF_93.8_/GO_93.8_ and EF_70_/GO_70_, respectively. Comparable distributions are observed for both types of flakes, with the doubly populated clusters being dominant. At high water content (Figure 6a), apart from the doubly occupied clusters, a considerable probability for clusters that include up to four flakes can be detected. As the water content drops, the distributions become broader, but clusters including up to four flakes (irrespective of the flakes’ orientation) still contribute significantly. Distances between pairs of sheets that constitute the clusters, D_c_, are also calculated and the corresponding probability distributions (P(D_c_)) are presented as insets in Figure 6a,b, respectively. Since these insets represent the distribution of inter-flake separations within a cluster, the maximum separations detected are those imposed by the cutoff criterion.

From the above clustering analysis, it follows that the EF-based systems form a larger number of low-populated clusters (i.e., mostly composed of two to four flakes) either at low or high water content conditions. For the GO-based flakes, although the doubly occupied clusters are the more frequent, a significant percentage of flakes participate in larger clusters with a more compact structure (see that the distributions in the insets are shifted to lower distances compared to their EF-based analogs).

#### 2.2.2. Effects of Water Concentration on the Morphological Properties of the Dispersion

To examine in more detail the way that the water concentration affects the tendency of the flakes to aggregate, we analyzed a series of different concentrations (see Table 2) for the most commonly used GO-based systems. Because of the constricted environment experienced by the flakes at low water contents, significant changes in their relative spatial arrangement are rather rare (see also the discussion in Section 2.2.3). Therefore, in order to improve the statistics, for these systems (i.e., GO_50_-GO_10_), runs starting from three different initial configurations were performed. Results for the average distance (D) between all pairs of flakes from each individual run together with the corresponding averages are depicted in Table 3. The average distance between graphene flakes at systems with high water concentrations (i.e., in systems GO_93.8_, GO_91.5_, and GO_86.7_) is found to decrease with decreasing water content, as one would expect, resulting in more compact structures. However, this trend is discontinued at considerably lower water contents (i.e., in systems GO_50_-GO_10_), where the average separation between flakes shows a tendency for stabilization. This behavior should be correlated with the formation of dynamically arrested states, as will be discussed in Section 2.2.3.

**Figure 7:**
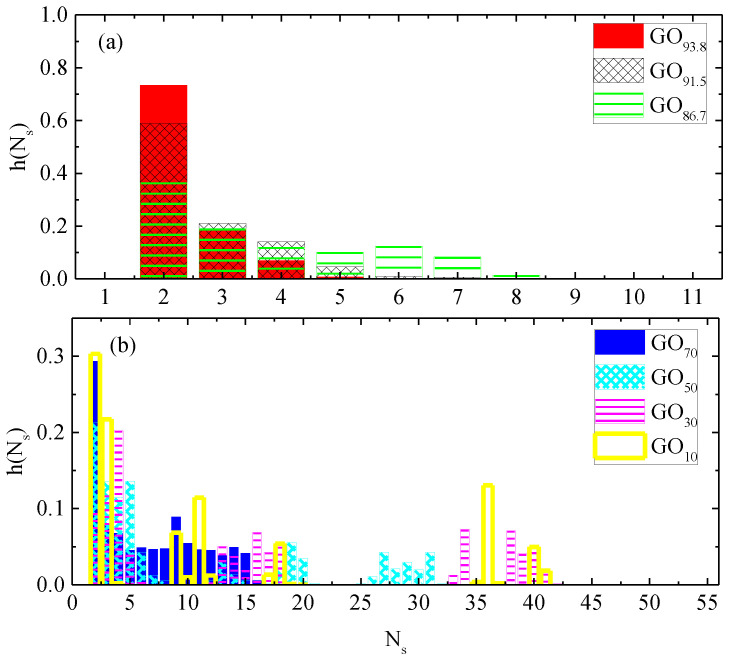
Probability distribution of the number of graphene flakes (N_s_) in a cluster characterizing the GO systems at high (**a**) and low (**b**) water content.

Insomuch as the average distance between flakes depends on concentration, the morphological characteristics of the formed clusters, such as the clusters’ occupancy (N_s_), should vary as well. As depicted in Figure 7a, the probability for the formation of small-sized clusters is higher at more dilute dispersions. When the flakes’ concentration increases (but still remains below the limit at which the average distance between the flakes is stabilized), so does the probability for the formation of clusters larger in size. Above this limit, the cluster size distribution becomes more widespread, with no systematic variation in the qualitative features of the clusters (Figure 7b). A common characteristic in all these systems, as noted when comparing systems GO_93.8_ and GO_70_ (Figure 6), is that the doubly populated clusters are the most abundant, while the formation of clusters consisting of up to approximately three to four flakes becomes very probable. If we take into account that the closest separation between the GO flakes at low water content is ~6.5 Å (see Figure S2) and that the separation up to which a tendency for parallel orientation can be detected between the GO flakes is ~20 Å (see Figure S4), it follows that in the case of clusters comprising almost parallelly arranged flakes, these should contain on average three flakes.

Therefore, such aggregates may well correspond to clusters with stacked flakes, in reasonable agreement with the experimentally determined stacked-GO cluster occupancy of approximately four, as was described in Section 2.1

A factor that is known to contribute significantly to the association between the flakes, and thus to the final microstructure of the dispersions, is hydrogen bonding [10,13]. To examine its role in this process, we monitored the extent of hydrogen bond formation for all systems, as listed in Table 4.

The detection of hydrogen bonding was based on geometric criteria, namely the maximum distance between the hydrogen donor and the acceptor to be 0.35nm and the angle formed by the hydrogen–donor–acceptor triplet to be lower than 30° [63]. Following this procedure, inter-flake hydrogen bonds were detected only between the GO-based sheets. The effect of concentration on hydrogen bonding between the GO flakes appeared to be minor, up to the concentration of 70 wt% in water, as listed in the second column of Table 4. For better statistics in the systems corresponding to lower wt% in water, the values shown represent averages over three independent runs from different starting configurations.

The observation that no hydrogen bonds are detected between the flakes of the EF-based systems should be attributed to the fact that the hydrogen atoms present at the periphery of the flakes are connected to carbons rather than to strongly electronegative atoms, so the C-H bonds are not polar enough to serve as hydrogen bonding donor sites. In contrast, GO-based flakes can dispose of hydrogen donors due to the presence of hydroxyl groups. The level of hydrogen bonding in the GO-based systems appears to grow upon an increase in the concentration of the GO flakes; this can be ascribed to the fact that the approach between the flakes creates the opportunity for more frequent interactions between their hydrogen donors and acceptors, thus increasing the probability of hydrogen bonding.

#### 2.2.3. Effects of the Oxidation Pattern and the Water Concentration on Dynamic Properties

As demonstrated in Section 2.2.2, the water content affects the final morphology of the dispersion by influencing the relative arrangement of the flakes and the size distribution of the formed clusters. Apart from the kinetics of aggregation, water concentration is also expected to affect the dynamics of cluster reformation at equilibrium conditions, provided that the oxidized flakes retain a sufficient level of mobility and are not trapped in a motionally arrested state [60]. At the examined temperature and pH conditions, between the limit of free diffusional motion of the flakes and the dynamically arrested state, there should be a concentration (or a narrow window of concentrations in case of frustrated systems) below which the dispersion enters a non-ergodic state [64].

**Figure 8:**
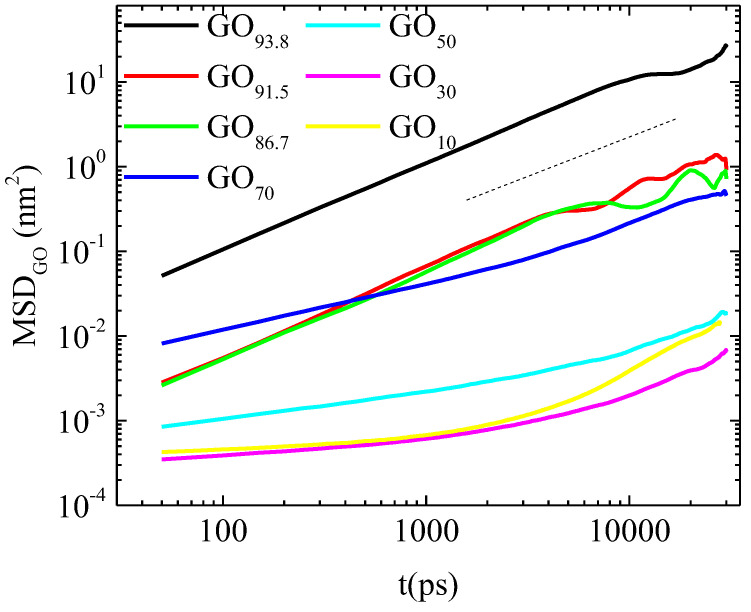
MSD of the geometric centers of GO sheets as a function of time, for GO-based systems of different water content (see Table 2). The straight dashed line denotes a slope of 1.

To determine the concentration below which a graphene oxide dispersion enters such a state, we examined the diffusional motion of the flakes at concentrations ranging between 93.8 and 10 wt% in water for the GO-based systems (see Table 2). Figure 8 depicts the mean squared displacement (MSD) for the geometric centers (gc) of the flakes as a function of time, for the GO-based systems.

A considerable slowing down is observed when the water concentration decreases, but the character of the gc motions remains practically diffusive at concentrations above a water content of 70 wt%. At this concentration, a marked change of slope in the MSD curve is observed even at short timescales, indicating a sub-diffusive motion. Below this water concentration, a further flattening of the MSD curves implies a constrained motion consistent with the so-called caging effect [65], i.e., the restricted motion of a particle (here the gc of a flake) within a transient “cage” formed by its slowly moving or frozen-in immediate neighbors. A similar analysis was performed for the EF_93.8_ and EF_70_ systems (see Figure S5). Similarly, the low-water-content system EF_70_ (i.e., at 70 wt% in water) exhibits a sub-diffusive behavior at the examined window.

The origin of this behavior at such water contents can be attributed to both geometric and energetic reasons. From the geometric point of view, if we consider each flake in the GO-based system as a platelet with a half-width *H* of approximately 1.5 Å (due to the presence of oxidized groups in both sides of the GO flake) and with an effective “diameter” 2 *R* twice its radius of gyration (which is calculated to be close to 16 Å), then any system with a volume fraction higher than φ_c_ = 0.96 × *H*/*R* = 0.09 should experience some correlations between the orientation of neighboring particles [66]. If we consider that each GO flake occupies approximately a volume of 3.2 × 4 × 0.3 nm^3^ = 3.84 nm^3^, then the volume fraction of this system amounts to 0.091 which is very close to the limit for the emergence of the orientational correlations (i.e., the beginning of restricted rotation). Systems GO_50_, GO_30_, and GO_10_ assume even higher volume fractions. A similar calculation for the EF flakes renders a value of φ_c_ ≅ 0.03 while the volume fraction of EF_70_ is approximately 0.05, i.e., already higher than the corresponding φ_c._

From the energetic point of view, any interaction that can promote the formation and stability of the clusters is expected to affect their diffusional motion. The more stable the clusters, the higher the effect on the degree of orientational and translational diffusion (the timescales for the diffusion and reorientation of clusters are longer compared to those of individual flakes). Since hydrogen bonding does not contribute to the self-association of the EF flakes as discussed earlier, this should be driven by van der Waals and electrostatic forces. For graphene-based flakes with a very low degree of oxidation (or for non-oxidized flakes), it is known that the van der Waals forces are responsible for an effective attraction between the aromatic rings of the flakes (reflecting the π-π* interactions) [67] and for their higher hydrophobicity when compared to flakes with a higher oxidation level. Therefore, these forces are expected to contribute to the self-association of the EF flakes and to play only a minor role in the more oxidized GO flakes. The remaining driving forces for the association of the flakes should therefore be of electrostatic origin.

In our case, both kinds of the oxidation patterns examined at neutral pH conditions result in a negative net charge for each flake (see Table 1). Therefore, any Coulomb-based mechanism participating in their self-assembly should involve at least partial screening of these charges by the counterions. The role of counterions in the effective attraction between like-charged colloids or electrolytes (similarly to our case of the likely-charged flakes) has been extensively discussed in the literature [68,69,70]. Ιt has been demonstrated that the counterions play an important role in this process through a dynamic balance between a strongly adsorbed and a loosely associated ionic layer; the latter can be “shared” between neighboring likely-charged particles providing a stabilization mechanism of the formed aggregates [71,72]. The dynamic characteristics of these layers may also significantly affect the conductive properties of the electrolyte dispersions [73,74]. To obtain information on the dynamic behavior of the counterions in our systems, their diffusional motion was monitored, and the influence of the different water content and oxidation pattern on the observed behavior was investigated. Moreover, the presence of a certain degree of dynamic heterogeneity, indicative of the formation of ionic populations with different mobilities in our models, was examined.

**Figure 9:**
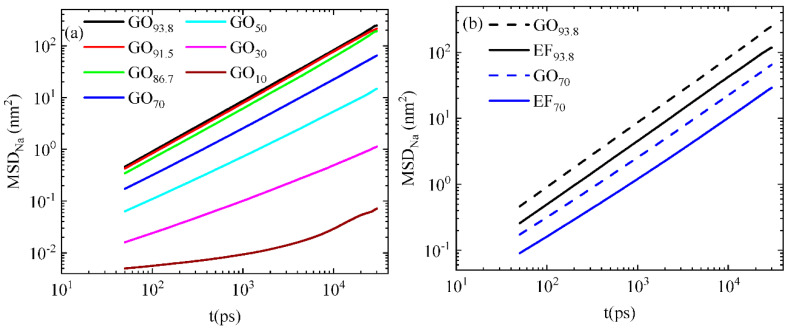
MSD of the Na^+^ ions as a function of time (**a**) for the GO-based systems at different water contents (**b**) for two of the GO-based and the two EF-based systems at the same water content.

The MSD of the Na^+^ ions is presented as a function of time in Figure 9a.

A gradual slowing down of the motion of ions is observed with the decrease in water content, in line with the behavior observed in the diffusion of the GO flakes. A considerable decrease in the MSD of the counterions is observed in the system of 70 wt% in water content, i.e., at the concentration at which the GO flakes enter their sub-diffusive regime (Figure 8). Comparing the motion of the counterions in the EF to that of the GO-based systems at constant water content (Figure 9b), it appears that the diffusion of ions is slower in the EF case. This lies in accordance with the slower diffusion of the EF flakes when compared to that of their GO analogs at the same concentration (see Figure S5). This implies a significant degree of coupling between the charged flakes and the counterions.

A parameter that can quantify the degree of motional coupling between the Na^+^ ions and the flakes is the residence time of the counterions close to the oppositely charged sites of the latter [75,76]. Since a counterion can dynamically associate or dissociate with a charged site, a way to monitor this process is by examining a characteristic correlation function *h*(*t*), defined as $h\left(t\right)=\frac{\langle c\left(t\right)c\left(0\right)\rangle }{\langle c^{2}\left(0\right)\rangle }$, where *c*(*t*) takes a value of 1 if the separation between two atoms remains shorter than a specified distance (i.e., they form a pair) at time *t*, provided that they formed a pair at time *t* = 0, and 0 otherwise. The characteristic distance necessary for the evaluation of *c*(*t*) is the one defined by the first minimum of the pair correlation function between the counterions and the charged carboxyl oxygens of the flakes (see Figure S6), i.e., 3Å in our case. Examination of *h*(*t*) may also provide information regarding the effect of water content on the degree of motional coupling between the counterions and the flakes.

**Figure 10:**
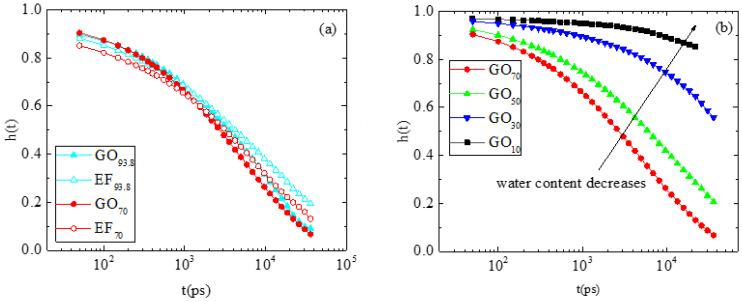
Comparison of the residence correlation functions describing the association of the sodium/carboxyl-oxygen pair in (**a**) two pairs of EF and GO-based systems at the same water concentrations and (**b**) GO-based systems at different water contents.

Figure 10a compares the residence pair correlation functions of the GO_93.8_ and EF_93.8_ (93.8 wt% in water) and the EF_70_ and GO_70_ (70 wt% in water) systems. Although no complete relaxation of *h*(*t*) is observed within the examined window (thus making the calculation of the residence time rather vague), it is clear that in both concentrations examined, *h*(*t*) decays slower in the EF-based systems, indicating a stronger coupling between the counterions and the flakes. In addition, as shown in Figure 10b, decreasing the water content to 70% wt and below increases the coupling strength between the counterions and the carboxyl oxygens of the flakes (since the decay rate of *h*(*t*) drops). These effects cannot be attributed solely to electrostatic origins, since the same atomic pairs are examined in all systems, irrespectively of the oxidation motif of the flakes or of the water content. We must therefore assume that entropic reasons related to the counterions should play a significant role as well.

**Figure 11:**
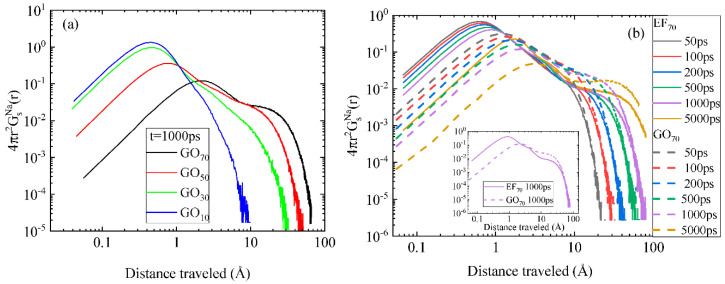
Self van Hove functions of the sodium counterions (**a**) in the lower-water-content GO systems at t = 1000 ps and (**b**) for the 70% wt% in water, EF_70_ and GO_70_ systems. The inset compares the van Hove curves corresponding to the EF_70_ and GO_70_ systems, at t = 1000 ps.

Previous works in polymer electrolyte systems [73,77,78] have demonstrated that depending on the physicochemical environment, populations of counterions with distinctly different mobilities may form, thus affecting their translational entropy. To examine whether the different oxidation motifs of the flakes or the different water content impart populations of counterions with different mobilities, the van Hove space–time correlation function was calculated [79]. The self-diffusion part of the van Hove function is given by $G_{s}(r,t)=\frac{1}{N}\sum_{i=1}^{N}\langle \delta \left[r-\left|r_{i}(t)-r_{i}(0)\right|\right]\rangle$, where *r_i_* represents the position of the counterion *i* at time *t*, *r* is the distance between the positions of the *i*th counterion at time *t* and at time 0, *N* is the total number of counterions, and *δ* is the Dirac distribution. The ensemble average is calculated over all configurations and time origins. This function is proportional to the probability that a Na^+^ is at position *r* at time *t*, given that the same Na^+^ was at the origin at *t* = 0. Figure 11a presents the self van Hove functions for the counterions in the GO_70_-GO_10_ systems, at a constant time interval but varying water content. A first observation is that as the water content decreases, the curves are shifted to shorter distances, in line with the behavior of the average diffusive motion of the counterions as shown in Figure 9a (a similar behavior is observed when comparing the spectra corresponding to the two different concentrations of the EF-based systems, as can be seen in Figure S7).

The additional information from these spectra is the appearance of more than one “shoulders”, indicating the separation of sodium ions into populations that have traveled different distances at the same timescale, i.e., they possess different mobilities. The peak appearing at shorter distances corresponds to the population that experiences a more restricted motion and can thus be related to the strongly adsorbed ions (termed henceforth as the “bound” population). The “shoulder” at longer distances should therefore be correlated with counterions that are more loosely bound to the oppositely charged sites of the flakes, thus exhibiting higher translational freedom. Another observation is that the distance traveled by the “bound” population stops shifting to shorter values when the water content becomes 30 wt% or lower (i.e., in the GO_30_ and GO_10_ systems). In other words, at very low water content, the contribution of this population to the overall translational entropy of the counterions remains constant. Accordingly, the ionic conductivity is expected to exhibit an analogous stabilization at water concentrations of 30 wt% or lower. Moreover, as the water content decreases, the amount of more mobile counterions decreases, and concurrently, the number of “bound” ones increases.

Figure 11b compares the self van Hove functions of the counterions in the EF_70_ and GO_70_ systems at different time intervals. A visual inspection of the curves reveals that the EF spectra are shifted to shorter distances compared to those describing the GO systems, in accordance with the average behavior noted in Figure 9b. Another remark is that at a constant time interval, the “loosely” bound ion population has practically traveled the same distance, irrespective of the oxidation pattern of the flakes. This is more clearly shown in the inset of Figure 11b. Consequently, the difference in counterion dynamics in the systems bearing the EF or the GO flakes arises solely from the behavior of the “bound” ion population. The same observation holds when comparing EF and GO systems at higher water contents (see Figure S8). This explains the findings that the average dynamics of counterions is strongly correlated with the flakes’ diffusional motion and that the longer residence time was observed in the EF systems. It also implies that any difference in ionic conductivity between systems bearing flakes comparable in size but with a different oxidation pattern would probably originate from the difference this pattern incurs in the percentage of the physically adsorbed ionic moieties.

**Figure 12:**
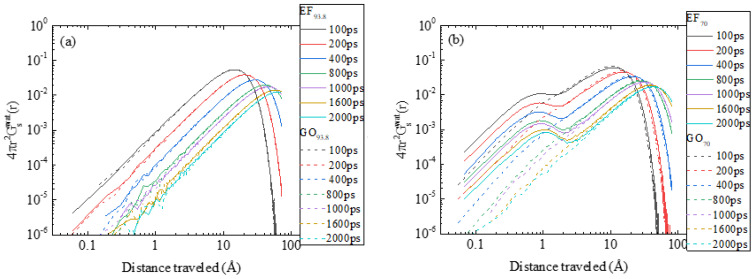
Comparison of the self van Hove functions of the centers of mass of water molecules in (**a**) 93.8 wt% and in (**b**) 70 wt% in water, at different timescales.

Apart from the effects of the oxidation pattern on the dynamics of counterions, the different morphological characteristics between the EF and the GO-based dispersions may also affect water dynamics. Figure 12 compares the self van Hove functions of the water molecules in the EF and the GO systems at 93.8 and 70 wt% water content.

At the higher water content (Figure 12a), the spectra practically coincide. At a lower water content (Figure 12b), the EF and the GO spectra overlap only in the region of the “fast” peak (i.e., the location of the maximum and the shape of the peak at longer distances), which can be ascribed to the freely diffusing molecules. However, a marked disparity between the spectra of the two models is observed at shorter distances. In the case of the EF-based system, a distinct peak can be observed at short distances, indicating the presence of a population of water molecules with considerably lower mobility. In the case of the GO system, only a weak “shoulder” can be observed at the leftmost part of the spectra.

For both systems, formation of hydrogen bonds between water molecules and the flakes is present at comparable levels (see Table 4). Therefore, the higher intensity of the low-mobility peak observed in the EF spectra must be attributed to another origin, such as to a small number of water molecules entrapped in between the closely packed flakes observed in the EF-based systems. The assignment of the low-mobility peak to a population of spatially constricted water molecules is corroborated by the fact that a similar peak can clearly be discerned at lower water contents in the GO-based systems as well (see Figure S9). At such low water contents (and higher GO concentrations), the translational motion of water becomes also sub-diffusive (see Figure S10).

## 3. Discussion

Analysis of the static and dynamic behavior of the systems studied reveals that both the oxidation motif of the graphene flakes and the water content decisively affect the morphological characteristics and the motional properties of different moieties in the dispersions. The absence of the in-plane oxygenated groups in the EF flakes allows the formation of closely packed (i.e., stacked) configurations with interlayer distances close to 5 Å, even at high water contents. The closest separation between the GO flakes is somewhat higher due to the presence of the in-plane oxidized groups. The latter, at the low-water-content limit, is stabilized at an average value close to 6.5 Å, in good agreement with the experimental value.

In the EF case, since there is no hydrogen bonding between the flakes, the close proximity between the like-charged flakes, appears to be facilitated by the electrostatic screening provided by the sodium counterions, the higher percentage of which remains strongly bound to the oppositely charged peripheral carboxyl groups. A similar electrostatic screening by the counterions is also present in the GO flakes; however, in this case, the residence time of the “bound” counterion population is shorter. Therefore, the local electrostatic screening provided by the counterions is not as efficient. A possible explanation for this behavior can be provided by considering the enthalpic/entropic interplay in each system. In the case of the EF flakes, a stronger coupling of the counterions to the oppositely charged carboxylate ions can be favored (at the expense of a higher entropy loss of the counterions), due to the enthalpic gain provided by the π-π interactions between the flakes. In the case of the GO flakes, the coupling of the sodium counterions with the carboxylate ions, although weaker compared to that present in the EF flakes, may allow for a spatial approach between the GO flakes sufficient for the formation of inter-flake hydrogen bonds. This results both in a hydrogen-bond-related enthalpic gain and in a lower loss of translational entropy of the counterions.

This counterion-driven partial “neutralization” of the flakes of both motifs also allows for the formation of clusters larger in size. At high water content, cluster sizes comprising two to four flakes are the most common in both oxidation motifs. In the EF case, the flakes within the formed clusters (at least at the examined water contents) appear to assume a parallel orientation only at the doubly occupied clusters (see Figure 4 and Figure 5). In the GO case, the separation between almost parallelly aligned flakes can be larger. At lower water contents, the distribution of the GO-based clusters becomes wider, but the formation of clusters comprising up to four flakes remains very frequent; it also appears very probable that the flakes within such clusters assume an almost parallel orientation, in line with the experimental findings. Another effect related to the oxidation motif is the possible entrapment of water molecules within the closely packed flakes. This effect appears to be more pronounced in the EF-based systems at intermediate water contents, due to the larger degree of restriction experienced by the water molecules within the stacked flakes. At lower water contents, restrictions in the translational motion of water molecules are manifested in the GO-based systems as well.

Water content affects considerably the physical properties of the dispersion in more than one way. For one, it increases the viscosity of the dispersion, resulting in a much slower diffusion of the flakes and consequently that of their electrostatically bound counterions. This in turn is expected to affect the ionic conductivity of the systems. It appears that the water concentration limit below which such kinetically arrested states develop relates to the volume fraction below which orientational correlations between the flakes sets in. Depending on the oxidation motif of the flake, other interactions (such as hydrogen bonding or π-π interactions) may also contribute to the increase in the degree of clustering, enhancing the orientational correlation effects between the flakes and thus shifting this limit.

## 4. Materials and Methods

### 4.1. Simulated Systems

We simulated two distinct, experimentally realizable oxygenated models of graphene, aiming at elucidating the role of the full oxidation versus edge oxygenation in the organization and in the dynamic properties of the oxidized graphene flakes in an aqueous environment and at neutral pH conditions. These two oxidation patterns are expected to bear quite different hydrophilicity levels, due to their different degrees of oxidation, while the location of the oxidized groups, either only in the periphery or both in the basal plane and the periphery, is anticipated to result in different characteristics of the aggregated structures. The EF model bears only carboxylate groups and hydrogens around the edges of the flake. Such a functionalization pattern can be fabricated by appropriate graphene oxide reduction procedures [80] and has been described in more detail in a past simulational work [45]. The second model is a fully oxidized graphene sheet with carboxyl, hydroxyl, and epoxy groups distributed on both surfaces as well as around the edges, according to the Lerf–Klinowski model [81]. More details, including certain self-assembly characteristics of this model in an aquatic environment but in the presence of other compounds as well, have recently been discussed [17].

**Figure 13:**
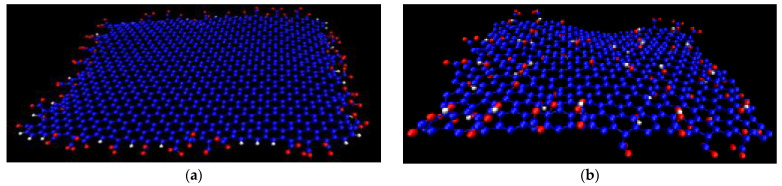
Graphene oxide sheets of the EF (**a**) and the GO (**b**) models. Oxygen atoms appear in red, hydrogen atoms in white, and carbon atoms in blue.

As a general case, for both the EF and the GO models, we considered two opposite zig-zag and two opposite arm-chair edges [82] following previous works [20,35,45,46,49,50,51]. Since no electronic properties are studied in this work, the alternating zig-zag/arm-chair motif of the functionalized edges is not expected to affect the key results regarding the driving forces for the spatial organization of the flakes. Figure 13 shows a graphical representation of the EF and the GO sheets.

The number of the oxygenated groups of each sheet, the relative proportion of each functional group, and the dimensions of the flakes are listed in Table 1.

Using the EF and the GO flakes, systems with different water content were constructed. For computational efficiency reasons, a smaller number of flakes were used for the systems with higher water content. For the GO sheets (which correspond to the most frequently experimentally realizable model [81,83]), a series of different concentrations were studied, by varying the amount of water contained in the simulation box in a systematic way between (10–93.8) wt%. For the EF flakes, two concentrations were examined, i.e., 93.8 and 70 wt% in water, which can be directly compared to the corresponding GO-based systems. Details of all the simulated systems are provided in Table 2.

In all systems, the carboxyl groups of the oxidized graphene flakes were taken to be deprotonated according to previous theoretical [20], simulational [38], and experimental [56] studies. In particular, for a system similar to a GO-based model, the degree of deprotonation close to neutral pH conditions was experimentally determined, as will be described in Section 4.3.

Initial configurations were created by randomly inserting both kinds of graphene flakes together with water molecules in the simulation box. An appropriate number of Na^+^ counterions were also added, in order to preserve the overall electrical neutrality.

The chemical composition of the EF and the GO sheets was kept constant throughout the simulations.

### 4.2. Simulation Methods

All-atom equilibrium MD simulations were executed using the GROMACS software [84], in the isothermal–isobaric (NPT) statistical ensemble. Pressure was kept constant at 1atm using the Parrinello–Rahman barostat, whereas the Nose–Hoover thermostat maintained the temperature constant at 300 K. The OPLS-AA forcefield [85] was used for the description of the energetic parameters for all graphene molecules; the TIP3P explicit solvent model [86] was used for water molecules. This forcefield/water model combination has previously been used for the parametrization of oxidized graphene models in an aqueous environment [51,55]. Periodic boundary conditions were invoked in all three directions. The particle mesh Ewald approximation was used for the electrostatic interactions [87]. After energy minimization, MD NPT runs of 100ns followed, using the last 40 ns of the trajectory (i.e., after equilibration) for the statistical data analysis. Multiple runs starting from different initial configurations were performed for systems where the motion of the graphene sheets was dynamically arrested, as it will be discussed later in the text.

### 4.3. Experimental System and Methods

Graphene oxide was experimentally synthesized utilizing a modified Hummers method [88]. The degree of oxidation was controlled by regulating the amount of the KMnO_4_ (here 0.5 g) that was used as the oxidation agent. More details on the synthesis, the morphological and the thermal characterization of the specific material, will be described in a separate work [57].

The degree of oxidation of the synthesized GO was determined by XPS measurements. The successful oxidation of GO was also verified by thermogravimetric analysis measurements that were performed utilizing a TA Instruments SDT600 TGA/DTA apparatus under inert Argon atmosphere; heating scans were recorded from room temperature to 700 °C with a heating rate 10 °C/min. Titration measurements were performed to determine the degree of ionization of the GO sheets. Initially, 10 μgr of each GO was diluted in 10 mL of water, and the dispersion was stirred for 1 h to assure homogeneity. Next, the appropriate amount of NaOH 1 M was added to raise the value of pH to 12. Finally, the titration curve was attained, and the pH values were recorded by adding HCl 0.1 M in steps of 20 μL, under continuous stirring.

The morphological characteristics of the synthesized GO were determined by X-ray diffraction (XRD). All measurements were performed utilizing a Bruker D8 Advance diffractometer in a diffraction angle range of 1.5–50° and a step of 0.01°/s. The CuKα radiation was used with wavelength *λ* = *λ*_CuKα_ = 1.54 Å. For layered materials that exhibit a periodic structure, such as the ones used in the current work, the measured XRD patterns show the characteristic (00*l*) diffraction peaks; the resulting spacing of the periodic structure is calculated using Bragg’s relationship, *nλ* = 2*d*_00*l*_sin*θ*, where *d*_00*l*_ is the interlayer distance, 2*θ* the diffraction angle, *λ* the wavelength of the radiation, and *n* the order of diffraction.

## 5. Conclusions

In this work, we employed atomistically detailed MD simulations to investigate the effects of the oxidation motif and of the water content, in the morphological and the dynamical aspects of graphene-based aqueous dispersions under neutral pH conditions.

Two different oxidation patterns were examined, i.e., either only edge-functionalized flakes with carboxylic groups or flakes with the presence of additional in-plane oxygenated groups, such as hydroxyls and epoxides. The water content ranged between ~93 wt% and 10 wt% (the lower water concentrations were examined only for the highly oxidized models). For one of the examined oxidation patterns, we compared simulation findings regarding key structural features of the formed aggregates, with experimental measurements on a system synthesized in a manner to resemble the simulated models. A good agreement was found between simulation results and experimental findings.

The oxidation pattern was found to affect the final spatial arrangement of the flakes, as well as the structural features of the clusters formed by them (relative orientation of the flakes, interlayer distance, distribution of cluster sizes). It also affected the degree of hydrogen bonding between the flakes, the dynamics of the water molecules that were entrapped within the aggregates, and the diffusive motion of the counterions.

Moreover, the water content played a decisive role in the diffusional motion of all the components of the dispersion. Below a certain water content (close to 70 wt% for the flakes of the examined sizes), the dispersions based on flakes of both oxidation patterns enter into a kinetically arrested state where the flakes become motionally restricted. Due to the strong coupling between the charged groups of the flakes and the counterions, average counterion dynamics is also significantly slowed down, while ionic groups of different mobilities are formed.

The above findings show that the graphene oxidation pattern, in conjunction with the water content of the dispersion, is a parameter that may lead to better control of key physical properties of such materials, thus offering new insight into their optimized use in modern industrial, environmental, and biomedical applications.

## Figures and Tables

**Table 1:** Description of oxidized graphene flakes.

Systems	Dimensions (nm^2^)	Composition of a Sheet		Net Charge Per Sheet (|e|)
Edge-Functionalized, EF	5 × 5	C COO^−^ H	1032 60 30	−60
		C:O 8.6:1		
Graphene Oxide, GO	3.2 × 4	C COO^−^ OH O	554 26 36 24	−26
		C:O 4.94:1 O(hydroxyl):O(epoxy) 3:2		

**Table 2:** Composition of the simulated systems. V_Box_ refers to average box dimensions.

Systems	No. of Sheets	%wt Graphene Concentration	%wt H_2_O Concentration	V_Box_ (nm^3^)	Na^+^
EF_93.8_	7	5.66%	93.8%	(14.46)^3^	420
EF_70_	56	27.48%	70%	(16.25)^3^	3360
GO_93.8_	7	5.76%	93.8%	(11.86)^3^	182
GO_91.5_	10	7.95%	91.5%	(11.96)^3^	260
GO_86.7_	10	12.42%	86.7%	9.55 × 11.8 × 9.55	260
GO_70_	56	28%	70%	(13.30)^3^	1456
GO_50_	56	46.7%	50%	(10.70)^3^	1456
GO_30_	56	65.4%	30%	(9.46)^3^	1456
GO_10_	56	84%	10%	(9.95)^3^	1456

**Table 3:** Average distance between all pairs of flakes for the GO-based models. Averaging among three independent runs was performed for the GO_50_, GO_30_, and GO_10_ systems.

Systems	<D> (nm)			
GO_93.8_	6.24			
GO_91.5_	5.71			
GO_86.7_	4.88			
GO_70_	6.03			
GO_50_	5.24	5.03	4.88	5.05 ± 0.18
GO_30_	4.77	4.42	4.30	4.49 ± 0.28
GO_10_	4.77	4.69	4.62	4.69 ± 0.07

**Table 4:** Hydrogen bonds between the oxidized graphene flakes (HBGG) and between the flakes and water molecules (HBWG), divided by the number of flakes.

Systems	HBGG/G	HBWG/G
EF_93.8_	0	246.3 ± 6.2
EF_70_	0	211.2 + 1.0
GO_93.8_	10.5 ± 0.5	207.8 ± 3.2
GO_91.5_	9.2 ± 0.4	207.9 ± 2.6
GO_86.7_	8.9 ± 0.4	202.7 ± 2.4
GO_70_	9.7 ± 0.2	189.0 ± 1.3
GO_50_	12.3 ± 0.8	167.7 ± 7.2
GO_30_	15.5 ± 0.2	127.5 ± 0.4
GO_10_	17.6 ± 0.5	56.0 ± 0.4

## Supplementary Materials

The following supporting information can be downloaded at: https://www.mdpi.com/article/10.3390/ijms232113459/s1.
