# Supplementary material for: The Role of Oxidation Pattern and Water Content in the Spatial Arrangement and Dynamics of Oxidized Graphene-Based Aqueous Dispersions
Source: Int J Mol Sci. 2022 Nov 3;23(21):13459. doi: 10.3390/ijms232113459 (PMC9653754; doi:10.3390/ijms232113459)
Supplement: Supplementary file 1 [file ijms-23-13459-s001.zip › ijms-1950934-supplementary.pdf]

Supporting Information

The Role of Oxidation Pattern and Water Content in the Spatial Arrangement and Dynamics of Oxidized Graphene-based Aqueous Dispersions

A. Rissanou[1,2,3], I. Karnis[3,4], F. Krasanakis[3] K. Chrissopoulou[3], K. Karatasos[1,2*]

1. Department of Chemical Engineering, University of Thessaloniki, P.O. BOX 420, 54124 Thessaloniki, Greece.

2. Department of Mathematics and Applied Mathematics, University of Crete, GR-71409, Heraklion, Crete, Greece

3. Institute of Electronic Structure and Laser, Foundation for Research and Technology-Hellas, 700 13 Heraklion Crete, Greece

4. Department of Chemistry, University of Crete, 70013 Heraklion Crete, Greece

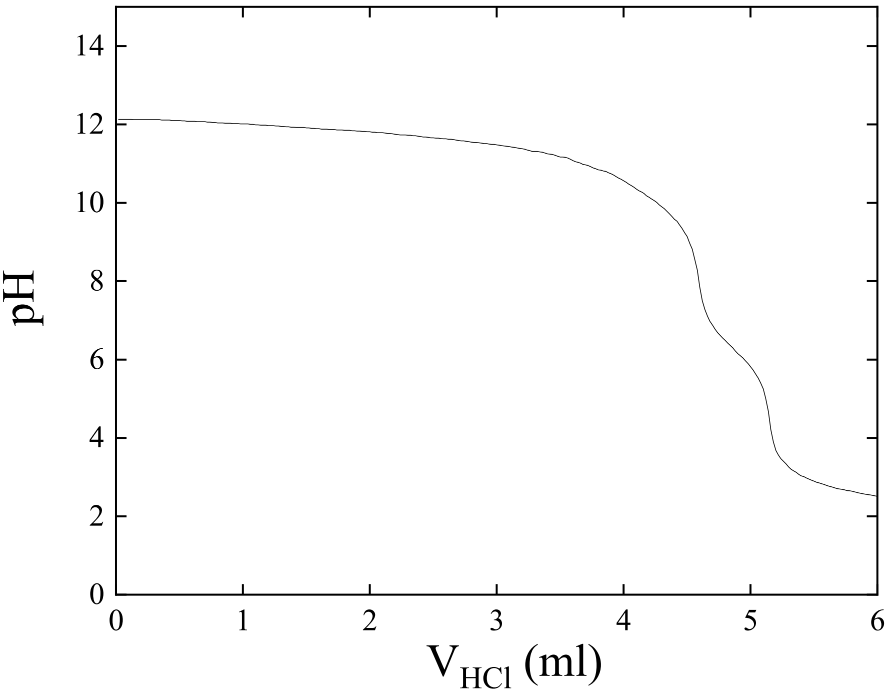

Figure S1: Titration curve of GO oxidized with 0.5 g of $KMnO_4$.

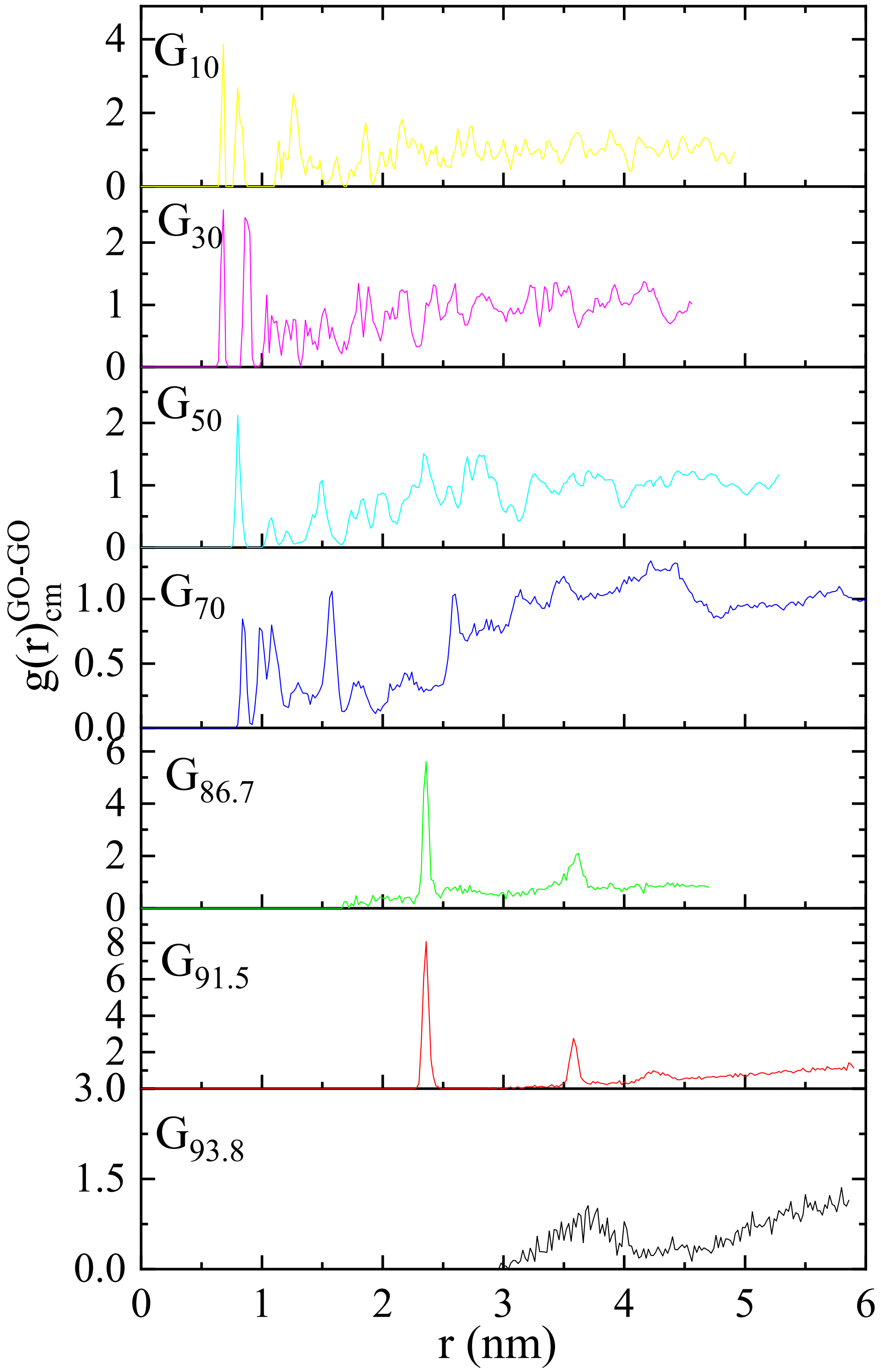

Figure S2: Radial distribution functions of the geometric centers of the GO-based systems.

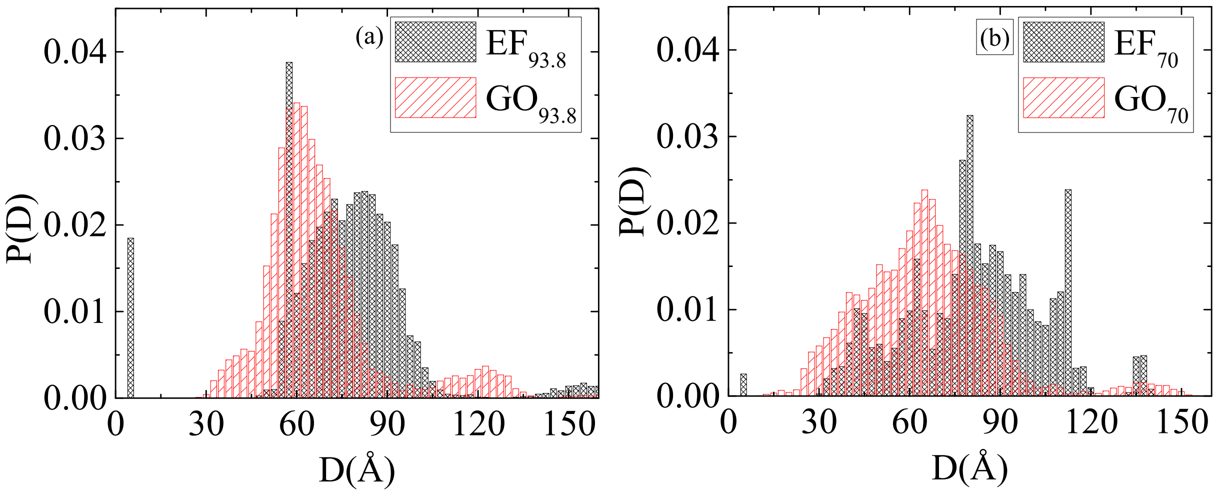

Figure S3. The probability distribution of the inter-sheet separation between all pairs of graphene sheets for the GO and the EF-based systems a) $EF_{93.8}$,$GO_{93.8}$ b) EF_{70},GO_{70}

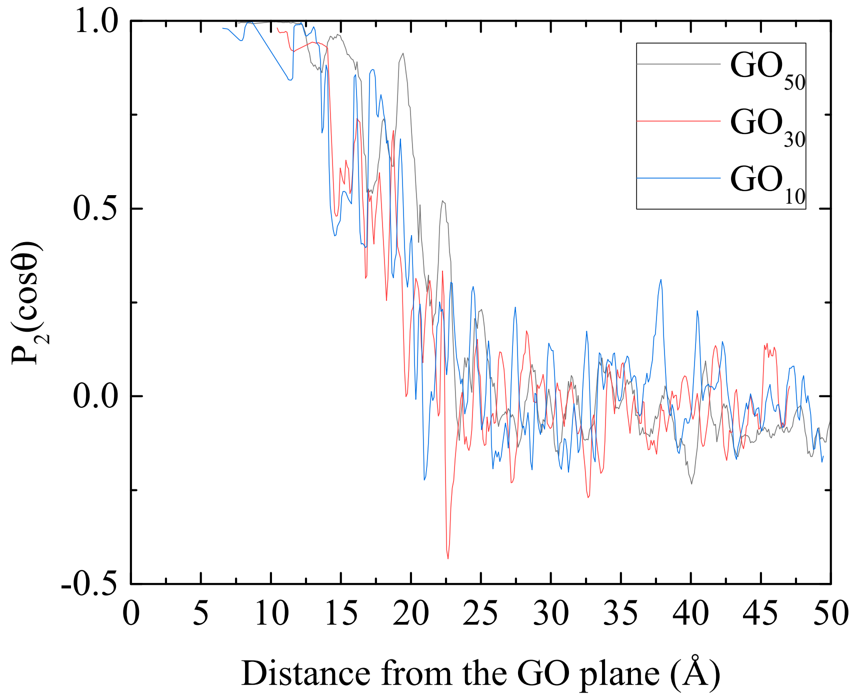

Figure S4: The orientational order parameter of the GO flakes as a function of their separation, for systems comprised by 56 flakes and at 50, 30 and 10 wt% water content.

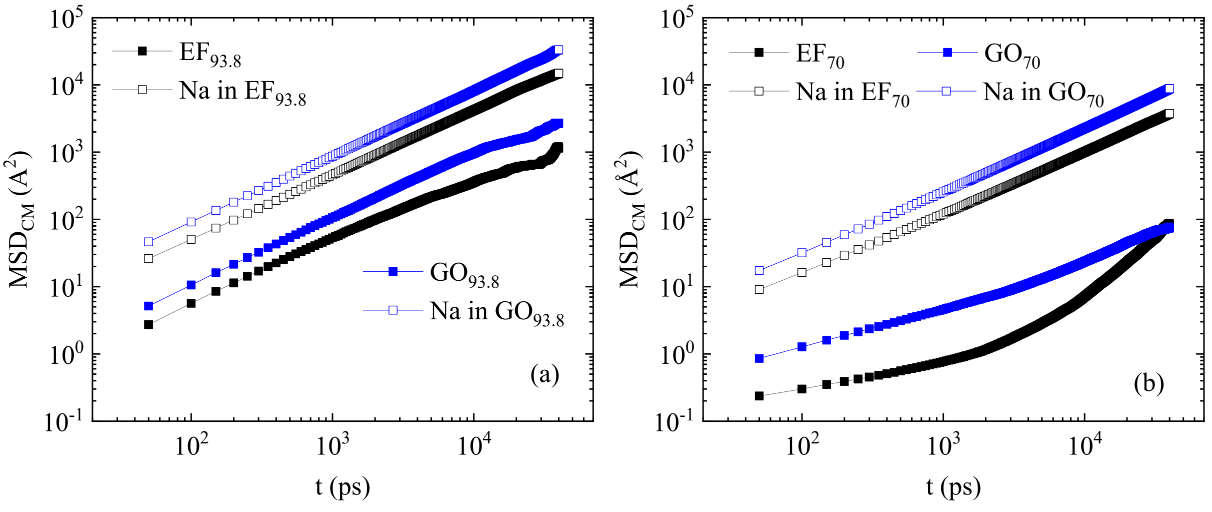

Figure S5: Comparison of the mean squared displacement of the sodium counterions and the center of mass of the EF and the GO flakes, at water contents : a) 93.8 % b) 70%

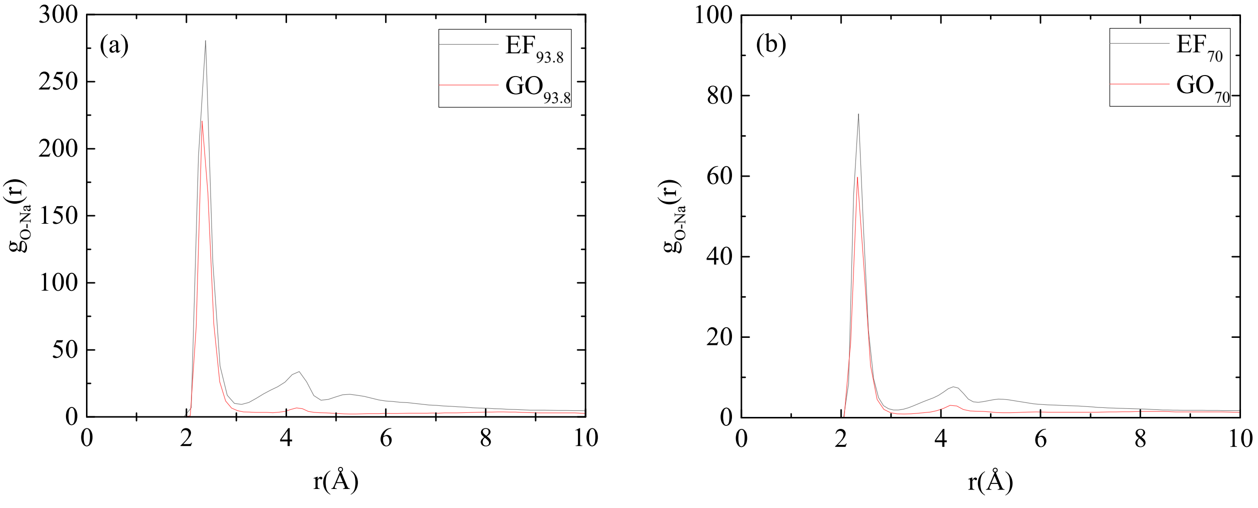

Figure S6: pair correlation functions between the sodium ions and the carboxyl oxygen of the flakes for the EF and the GO-based systems at water contents wt% a) 93.8% and b) 70%

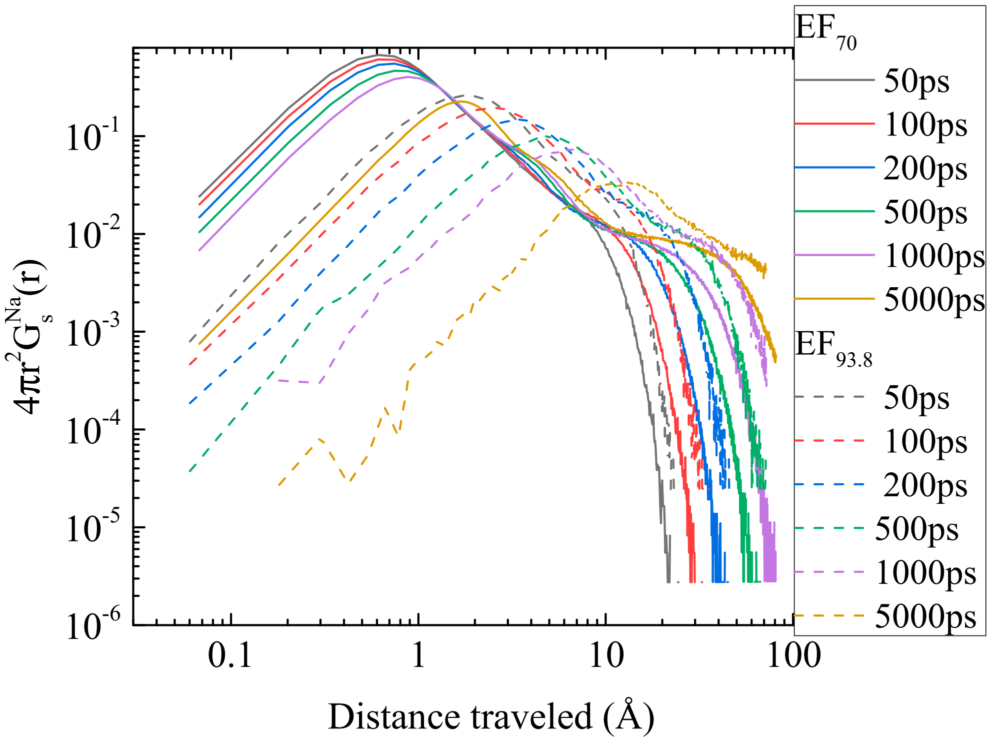

Figure S7: Comparison of the Self van Hove spectra of the Na+ counterions in the EF-based systems at different timescales.

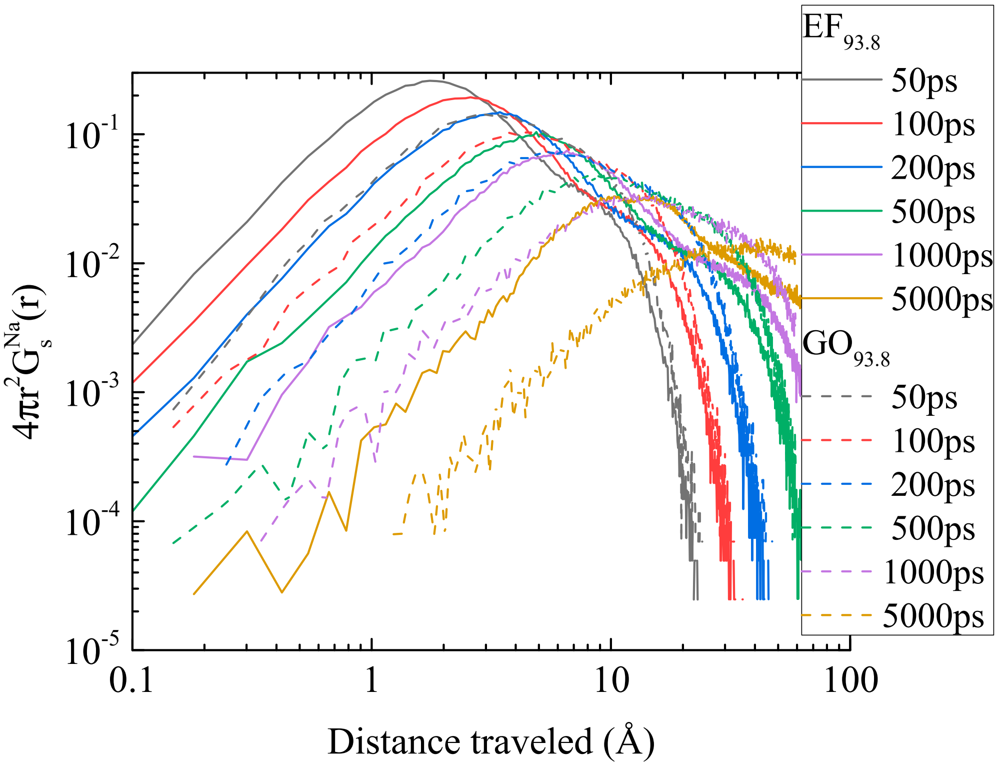

Figure S8: Comparison of the self van Hove functions of the Na+ counterions between the EF and the GO based systems at 93.8% wt% in water content

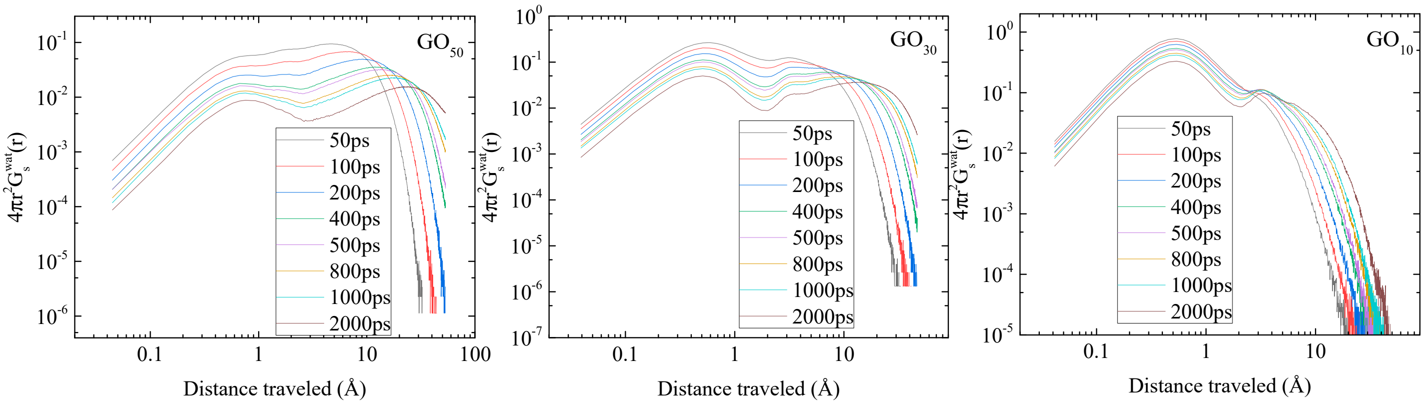

Figure S9: Self van Hove functions of the centers of mass of the water molecules for the lower water content systems of the GO-based models.

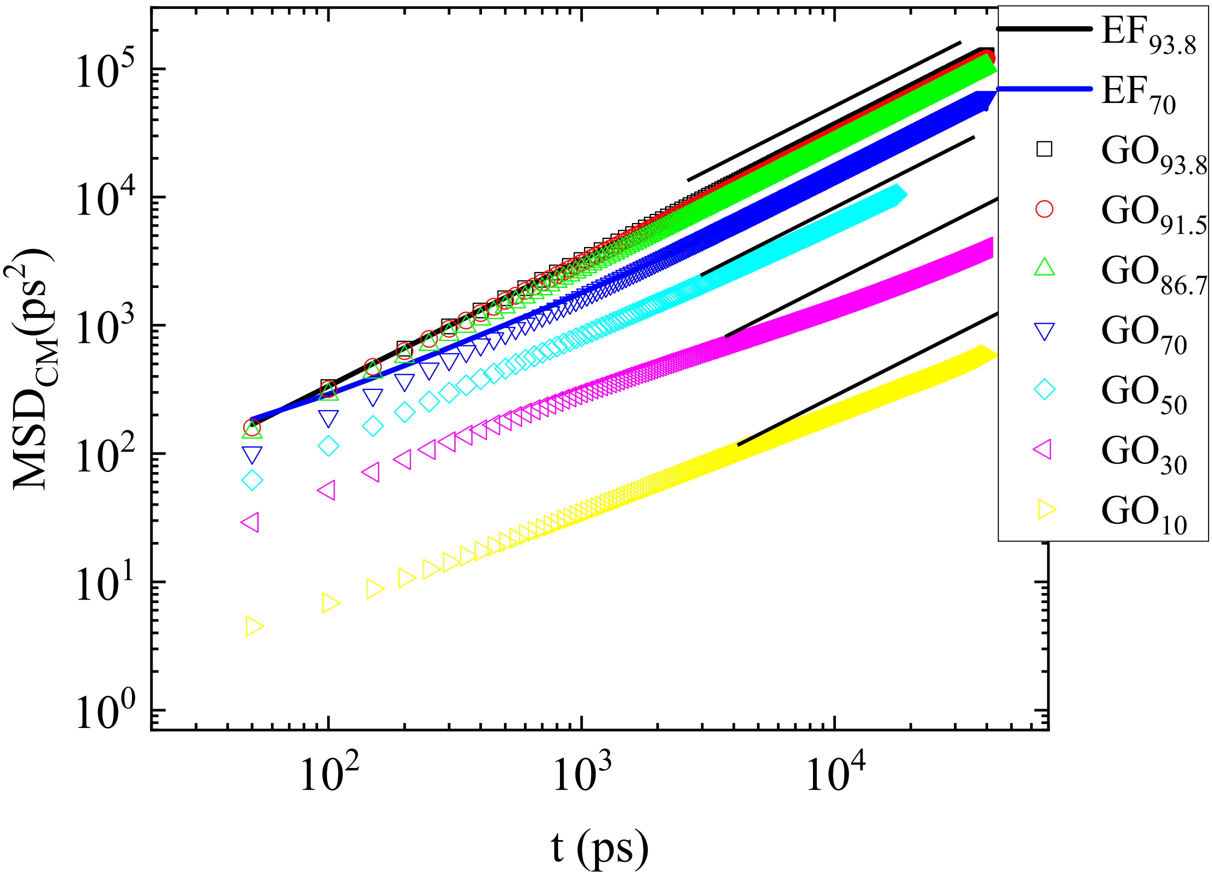

Figure S10: Mean squared displacement of the centers of mass of the water molecules for all the models examined. The short straight lines denote a slope of 1.
